# Shell properties of commercial clam *Chamelea gallina* are influenced by temperature and solar radiation along a wide latitudinal gradient

**DOI:** 10.1038/srep36420

**Published:** 2016-11-02

**Authors:** Francesca Gizzi, Maria Giulia Caccia, Ginevra Allegra Simoncini, Arianna Mancuso, Michela Reggi, Simona Fermani, Leonardo Brizi, Paola Fantazzini, Marco Stagioni, Giuseppe Falini, Corrado Piccinetti, Stefano Goffredo

**Affiliations:** 1 Marine Science Group, Department of Biological, Geological and Environmental Sciences, University of Bologna, Via F. Selmi 3, I-40126 Bologna, Italy, European Union; 2 Laboratory of Fisheries and Marine Biology at Fano, University of Bologna, Viale Adriatico 1/N, I-61032, Fano (PU), Italy, European Union; 3 Department of Chemistry “Giacomo Ciamician”, University of Bologna, Via F. Selmi 2, I-40126 Bologna, Italy, European Union; 4 Department of Physics and Astronomy, University of Bologna, Viale Berti Pichat 6/2, 40127 Bologna, Italy, European Union; 5 Museo Storico della Fisica e Centro Studi e Ricerche Enrico Fermi, Piazza del Viminale 1, Roma, Italy, European Union

## Abstract

Phenotype can express different morphologies in response to biotic or abiotic environmental influences. Mollusks are particularly sensitive to different environmental parameters, showing macroscale shell morphology variations in response to environmental parameters. Few studies concern shell variations at the different scale levels along environmental gradients. Here, we investigate shell features at the macro, micro and nanoscale, in populations of the commercially important clam *Chamelea gallina* along a latitudinal gradient (~400 km) of temperature and solar radiation in the Adriatic Sea (Italian cost). Six populations of clams with shells of the same length were analyzed. Shells from the warmest and the most irradiated population were thinner, with more oval shape, more porous and lighter, showing lower load fracture. However, no variation was observed in shell CaCO_3_ polymorphism (100% aragonite) or in compositional and textural shell parameters, indicating no effect of the environmental parameters on the basic processes of biomineralization. Because of the importance of this species as commercial resource in the Adriatic Sea, the experimentally quantified and significant variations of mass and fracture load in *C. gallina* shells along the latitudinal gradient may have economic implications for fisheries producing different economical yield for fishermen and consumers along the Adriatic coastline.

Organisms are able to modulate their developmental trajectory and alter gene-expression patterns in response to abiotic (such as temperature or photoperiod) or biotic (such as those emanating from predators, conspecifics or food) environmental cues^1^. Environmental parameters influence the organism, producing a non-pathological phenotype, appropriate for that environment. An enduring puzzle in evolutionary biology is to understand how individuals and populations adjust to changing environments. Intraspecific phenotypic variation is believed to arise from divergent selection pressures between different environments^2^, from environment-independent phenotype generation, as well as from potentially non-adaptive effects of the environment on phenotype^2^. Thus, a particular environment can elicit different phenotypes from the same genotype^1^. The ability of organisms to produce different phenotypes under different environmental parameters in natural populations is a critical issue to understand how species might face future changes.

Phenotype plasticity is the ability of an organism to produce a range of relatively fit phenotypes, by altering morphology, movement, behavior or rate of biological activity in response to fluctuations in environmental parameters^3^. Considering the existing effects of anthropogenic activities on the environment, organisms exhibiting higher phenotypic plasticity might cope better to broad scale disturbances, such as climate change^4^.

Calcifying marine organisms (e.g. corals, echinoderms and mollusks) are likely to be among the most susceptible organisms to changing environmental parameters^3^ and show morphological variations of the skeleton/shell related to bottom topography, sediment characteristics, hydrodynamic processes^5^, and especially pH and temperature^6^. These organisms make extensive use of calcium carbonate (CaCO_3_), one of the most abundant minerals in nature, as a structural and/or protective material through the biomineralization process^7^. Morphology, mineralogy and chemistry of biologically formed CaCO_3_ skeletons are largely dependent on both biology and environmental surroundings, with structural proteins and enzymes that act as keys to controlling internal conditions and that respond to external environmental parameters^8^. Mollusks are able to exert an exquisite biological control on the biomineralization process by determining which type of CaCO_3_ polymorph precipitates through the control of intra-skeletal macromolecules^8^. It is well known that the intra-crystalline skeletal organic matrix (OM) plays a major role in biomineralization and as in all biominerals, mollusk shells also contain OM that rarely exceeds 5% weight of the total shell^9^.

Environmental factors, such as solar radiation, food availability, oxygen, salinity and temperature, all influence energy expenditure in marine organisms, especially in temperate seas, where marine organisms show marked seasonal patterns in growth, reproduction and abundance. Solar radiation (SR) and sea surface temperature (SST), are widely used as monitoring parameters for ecological studies and generally influences the demography and skeletal properties of marine calcifying organisms, including sponges^10^ and non-zooxanthellate coral^11^,^12^. The effects of SST on calcification and growth have been deeply studied on several calcifying marine organisms, including mollusks^6^,^13^,^14^,^15^,^16^, but no study investigated the effect of SR on calcification and growth of bivalve mollusks.

To study the effect of SR and SST on marine organisms, latitudinal gradients are useful natural laboratories, influencing variations in SR and SST and allowing to examine long-term effects on populations of the same species, adapted to different environmental conditions^6^,^17^. Several studies on bivalves were performed along latitudinal gradients, focusing on biodiversity^18^, growth rate, body size and lifespan^15^,^16^, but no one investigated the shell variation at multiscale level. Mollusk shell morphology is particularly sensitive to environmental parameters, varying in relation to depth^19^, current^20^, wave exposure^21^, bottom type, sediment^19^,^22^, pH and temperature^6^. The quagga mussel *Dreissena bugensis* shows plasticity in shell morphology in relation to depth: deep ones presents a more laterally flattened shell and a more oval shape than those from shallow water habitats^19^. The clam *Mya arenaria* from sandy bottoms shows a longer and narrower shape, compared to a rounder shape when grown in gravel^22^. Shell shape of the limpet *Lottia gigantea* changes as a function of intertidal zonation and related environmental factors, such as resistance to desiccation, thermal stress and wave impact, by developing high spiraled and heavily ridged shells which may reduce the likelihood of reaching elevated body temperatures^21^. Growth in length and height of the shells of the cockle, *Cerastoderma edule*, cease in winter when mean water temperatures fell to 5 °C^14^. Bivalve growth is not very affected by water temperature variations between 10° and 20 °C, but decreases at low temperatures (below 10 °C) or high temperatures (above 20 °C)^13^. High temperature influences key processes that can impair calcification in bivalves, and together with food availability plays an essential role in mollusk shell growth^23^.

The observed phenotypic plasticity of many marine calcifying organisms in relation to environmental parameters makes them potentially ideal models for studying such plastic responses and associated trade-offs in the face of global climate change.

The clam *Chamelea gallina* (Linnaeus 1758) is a common infaunal bivalve of the Mediterranean Sea, where it inhabits well-sorted fine sand biocoenosis at 3–7 m depth and has a considerable economical relevance for fishery^24^,^25^. In the 1970s the development of clam fisheries based on hydraulic dredges led to an over-exploitation of the resource with a dramatic decrease in clam population density associated with a reduction in the number of clams over 25 mm long, the minimum legal marketable size, although the maximum length recorded for this species is about 50 mm^24^. In Italy, in the late 1970s the fishery yielded 80,000-100,000 metric tons, however currently it does not exceed 20,000 metric tons^26^. Recently, there is growing concern for the survival of bivalve communities because large inter-annual fluctuations in stock abundance, periodic recruitment failure and irregular mortality events threaten the biological and economic sustainability of this fishery, especially in the Adriatic Sea^25^,^26^. Thus, studies on this species are of critical importance for developing appropriate management strategies for one of the most important economic sectors of southern EU countries. The Food and Agriculture Organization of the United Nations (FAO) reports a mean annual total catch of about 60,000 tons (2004–2013) in the Atlantic, Mediterranean and Black Sea, with the largest catches in Italy (22,000 tons) and Turkey (33,000 tons).

Several studies demonstrated that changes in abiotic environmental factors, such as temperature, salinity and oxygen strongly influence immune parameters of *C. gallina*, making it more susceptible to infection and diseases^27^.Water temperature has a dominant role also in shell growth of *C. gallina*^25^,^28^. Temperatures below 10 °C strongly slow or inhibit shell growth, whereas values above 28 °C reduce energy absorption and increase energy expenditure via respiration, thus suppressing shell growth^25^,^28^. Calcification of *C. gallina* seems to be related to temperature and food conditions, showing widely spaced growth bands during winter-spring, while narrow growth increments are deposited in summer-autumn^25^.

Several studies have been reported on variations of shell biometry in response to environmental parameters^6^,^19^,^20^,^22^,^29^, however few studies comparatively analyzed shell features at the micro and nanoscale level along environmental gradients^30^,^31^,^32^,^33^. The present study aimed to investigate the phenotypic response to the environment of shell features of *C. gallina* at different scales of observation, in six populations along a latitudinal gradient.

## Results

### Environmental parameters

SR and SST both varied among sites (Kruskal–Wallis test, df = 5, and p < 0.001; Table 1) and correlated negatively with latitude (Supplementary Fig. S1). The Monfalcone site, located in the Gulf of Trieste, has a higher SST than typically expected for this latitude^34^.

### Shell biometric parameters

**Figure 1:**
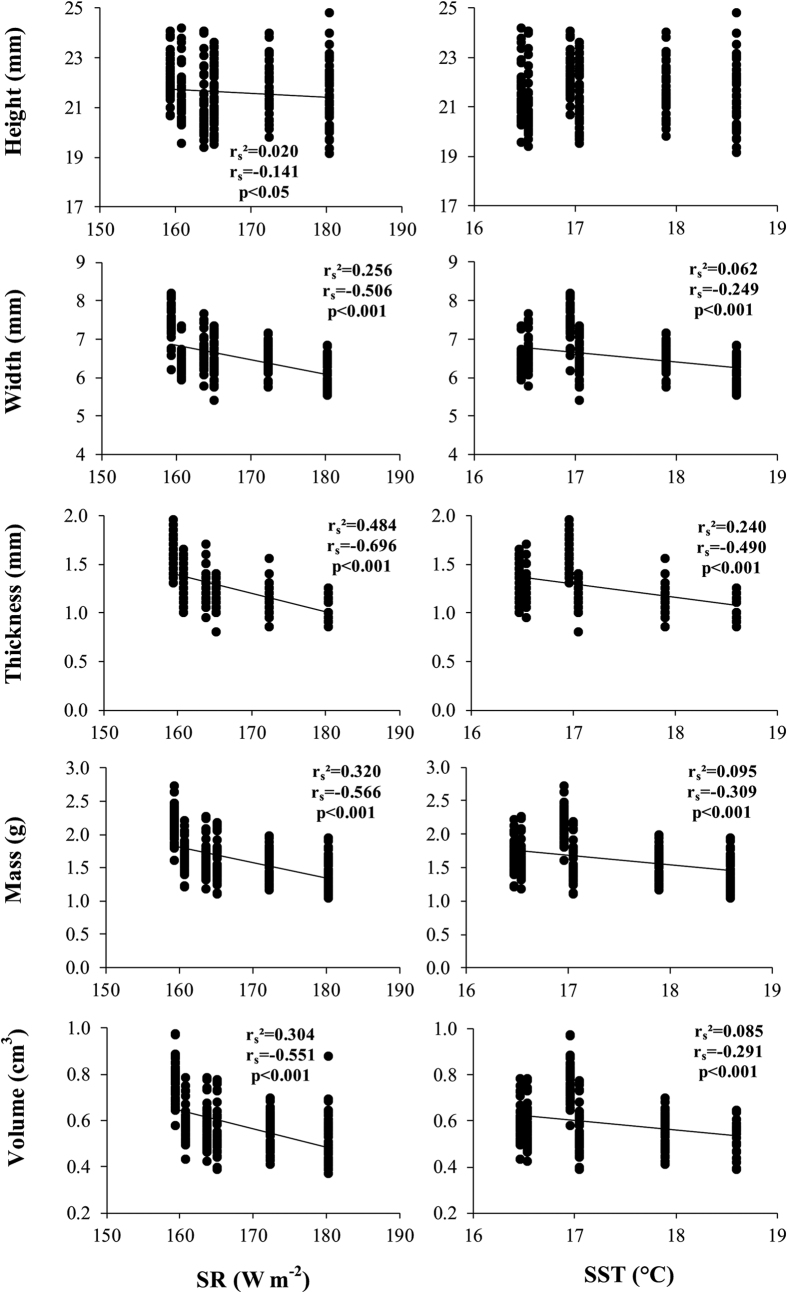
Shell biometric parameters. Macroscale level. Variation in the biometric parameters of *C. gallina* with environmental variables (SR and SST). r_s_ = Spearman’s determination coefficient. n = 40 in each population. Mean values for each population are listed in Table 2.

**Figure 2:**
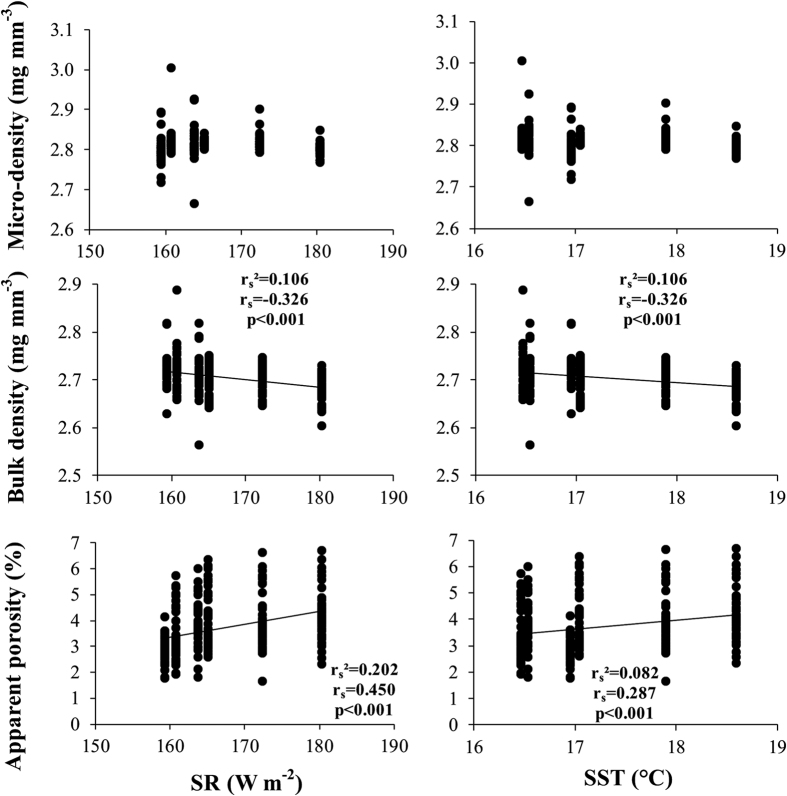
Shell biometric parameters. Macro- and microscale levels. Variation in the shell parameters of *C. gallina* with environmental variables (SR and SST). r_s_ = Spearman’s determination coefficient. n = 40 in each population. Mean values for each population are listed in Table 3.

Clam biometric parameters (length, height, width, mass, volume, micro-density, bulk density and apparent porosity) were homogeneous between left and right valves, thus data from both valves were pooled for following analyses. Since only clams of commercial size (25–30 mm) were considered in this study, shell length was homogeneous among populations (Table 2). Shell macroscale biometric data, height, width, mass and volume (with the exception of length), bulk density and apparent porosity were significantly different among sites, thus correlations analyses between SR or SST and clam parameters were performed (Tables 2 and 3; Figs 1 and 2). Micro-density did not differ among sites (Table 3). Width, mass, volume, correlated negatively with SR and SST, while shell height correlated only with SR (Fig. 1). Bulk density and apparent porosity correlated negatively and positively, respectively, with both SR and SST (Fig. 2). All parameters were more highly correlated with SR than with SST (Figs 1 and 2). Differences found in *C. gallina* shells among populations were related to tri-dimensional shell biometric parameters (height, width, mass, volume, bulk density and apparent porosity). To check if differences at bi-dimensional level occurred among populations, the bi-dimensional shell shape parameters (perimeter, area, aspect ratio, solidity, circularity and roundness), in addition with length and height, were analysed using multivariate statistical analysis (PCA). No differences were found among populations (see Supplementary Information; Supplementary Fig. S2a,b).

### Shell microstructure

The scanning electron microscopy observations (Supplementary Fig. S3a,b) showed that the microstructure of *C. gallina* shell contains two main layers, according to what observed in most venerids^35^. In the outer layer compound prisms are observed (Supplementary Fig. S3c). They are formed by the compact assembly of grains (Supplementary Fig. S3f). The inner layer is homogeneous and is formed of irregular granules (Supplementary Fig. S3e). Among these grains, having a size around 1 μm, layers of materials are dispersed (Supplementary Fig. S3h). A middle layer, which represents the transition zone between the inner layer and the outer one, is characterized by the presence of granules (Supplementary Fig. S3d), bigger than the ones observed in the inner layer (around 3 μm), from which the prisms start their structure. The granules observed in the outer and inner layer are quite similar in size and shape (Supplementary Fig. S3f,h). All populations revealed the same pattern at each considered magnification.

### Shell mechanical and mineral features

**Figure 3:**
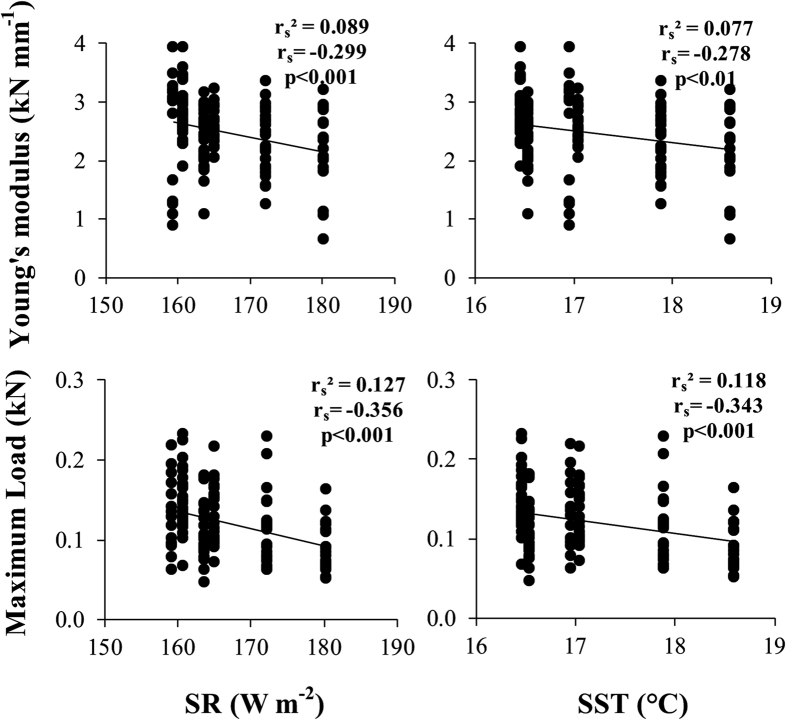
Shell mechanical properties. Variation in mechanical properties of *C. gallina* with environmental variables (SR and SST). r_s_ = Spearman’s determination coefficient. Samples number and mean values for each population are listed in Table 4.

Mechanical tests showed differences among populations for Young’s modulus and fracture load (Table 4), and both parameters correlated negatively with SR and SST (Fig. 3).

The analysis of the inorganic phase was obtained from the results of X-ray powder diffraction patterns (XRD) and Fourier transform infrared spectroscopy (FTIR) data. Both techniques showed that shells from all populations were composed of pure aragonite and no other mineral phase was detected (Supplementary Figs S4 and S5). Full width at half maximum (FWHM) calculated for the (111) peak of each diffraction pattern was different among populations but not correlated with SR and SST (Supplementary Table S1). In FTIR spectra the bands at 1484 cm^−1^ (*ν*_*3*_) 859 cm^−1^ (*ν*_*2*_) and 712 cm^−1^ (*ν*_*4*_), typical of aragonite, did not shift among samples (Supplementary Table S1; Fig. S5). The wavenumber of the *ν*_*2*_ band is a function of the content of Sr within the aragonitic sample^36^; accordingly a Sr content of about 8000 ppm can be estimated in each shell, independently from the collection site. A series of successive grindings of the powder from a shell sample for each population was carried out, following a reported procedure to generate the grinding curve, a qualitative estimation of the atomic order^37^. The bands heights were measured and the height ratios *ν*_*4*_/*ν*_*3*_ and *ν*_*2*_/*ν*_*3*_ were calculated. All the ratio values fitted in a curve in the *ν*_*4*_/*ν*_*3*_ vs *ν*_*2*_/*ν*_*3*_ graph (Supplementary Fig. S6). The obtained curve was compared with other curves fitted from data from different marine calcifying organisms (Phyla: Cnidaria, Anellida and Mollusca) and from geogenic and synthetic aragonite^37^ (Supplementary Fig. S6). The curve fitted from *C. gallina* data was located between those of geogenic and synthetic aragonite, along with the other biogenic aragonite samples (Supplementary Fig. S6).

The content of intra-skeletal OM, as weight percentage, was measured by thermogravimetric analysis (TGA; Supplementary Fig. S7). It was in average below 2% in all samples and homogeneous among populations (Supplementary Table S2); an equivalent result to that obtained from several other mollusk^38^.

## Discussion

The main aim of this study was to investigate the effect of SR and SST on shell features at macro (biometry), micro (texture) and nanoscale (atomic order and composition) levels, in natural populations of the common clam *C. gallina* along a latitudinal gradient in the Western Adriatic Sea, as a case study to gain further insight on the relationship between phenotype and environment in calcifying marine organisms. The possible relationships between growth rate and SR and SST were not considered in this study. Further investigation will be necessary to better understand how bivalves’ growth respond to changes in SR and SST.

At macroscale level, shells of the same length of *C. gallina* were affected by increasing SR and SST, presenting lighter, thinner, more oval shaped, more macro-porous and less resistant to fracture valves in the warmer and more irradiated populations. Previous studies show that the shells of many mollusks species are affected along latitudinal gradient due to decreasing SST^6^,^39^. A possible explanation could be that at low SST, CaCO_3_ is more soluble and seawater is less saturated, increasing energetic costs of shell formation^40^. Moreover, it was shown that low SST directly reduces growth^41^ and development^42^. Conversely, in this study *C. gallina* seemed to be negatively affected in shell biometry by high SST, showing an opposite trend. The effect of temperature on physiology of *C. gallina* has been studied in specimens exposed to different temperature by Moschino and Marin^28^. They investigated the scope for growth balancing the processes of energy acquisition (i.e. feeding, digestion) with the energy expenditure (i.e. respiration, excretion) and providing an instantaneous measure of the energy state^28^. Specimens of *C. gallina* exposed to high summer temperatures (28 °C), show a reduced energy absorption and an increased energy expenditure via respiration, negatively affecting the energy balance^28^ and probably growth, as found during summer season (temperature > 27 °C) in specimens from the eastern coast of Spain^25^. A drop in metabolism was recorded in the snail *Littorina saxatilis* exposed to elevated temperatures, with negative consequences in growth and fitness^43^. Moreover, oxygen depletion due to high temperatures may produce detrimental effects on the physiological performance of clams, as observed in the bivalve *Ruditapes decussatus*^44^. *C. gallina* seems to have relatively low tolerance to high temperatures in comparison with other bivalve species, showing a great influence in the overall physiological responses and heavy stress conditions when exposed to high temperatures, demonstrating that temperature could be a tolerance limit for this species^28^.

Despite *C. gallina* being an infaunal bivalve, all macroscale (biometric) parameters of this species seemed to be more correlated with SR than SST. SR could have no direct effect on this species, but the SR latitudinal gradient is related to other abiotic and/or biotic parameters not investigated in this study, such as phytoplankton density and its distribution. Food concentration is one of the major factors influencing the growth of suspension feeding bivalves^45^. SR affects the growth, survival and distribution of phytoplankton^46^, which represent the food source for higher trophic organisms, such as bivalves. Phytoplankton distribution in the Adriatic Sea is characterized by the relative influence of northern Italian rivers and by the influence of Mediterranean waters on the southern Italian coasts, showing a generally decreasing trend of nutrient concentration from North to South^47^. The northern Adriatic, influenced by Italian rivers outlets, was marked by low diversity but high density of phytoplankton; the southern Adriatic influenced by Mediterranean waters, exhibited high diversity but low density of phytoplankton^47^. The lower presence of phytoplankton density in southern Adriatic, could cause feeding deficits and consequently a reduction of available energy for clam to invest in shell construction, which could explain the reduction in weight, thickness and the higher porosity and fragility of shells.

Differences found in SST and SR along the gradient could also influence the type and/or density of predators. Predation is an important factor associated with morphological plasticity in bivalves, which can exhibit induced responses based on the capture techniques of the predators^48^. Bivalves’ principal defense is their strong calcareous shell, and among shell characteristics, thickness is the most influential factor for shell strength. The increased of bivalve shell thickness can thus reduce the success of many predators^49^. The blue mussel *Mytilus edulis* exposed to high predation density shows a thicker and more robust shell than those exposed to lower predation^49^. Similar results, showing an increase in shell thickness in response to predators, were found in several mollusks species^48^,^49^,^50^. Experimental works on gastropods show that shell thickening in response to predators could be partly due to avoidance behavior, resulting in lower growth rates partly due to direct result of increased shell deposition in the presence of predators^50^.

The observed phenotypic changes found in *C. gallina* could occur by genetic or plasticity variations, as found in other bivalves^48^, and could be explained by SST difference, nutrient concentration and/or density of predators along the latitudinal gradient. Increased porosity, decreased stiffness (Young’s modulus) and reduced thickness of *C. gallina* shells, with increasing SR and SST, lower the shell’s fracture load. Resistance to breakage generally increases as the square of the shell thickness increases^39^, and the breaking resistance doubles with an increase of 41% in thickness, thus providing a good return in shell strength for each unit of shell thickening^6^. The warmer and more irradiated populations of *C. gallina* showed reduced shell stiffness and fracture load, leading to a modified shell resistance, with a lower load fracture and damage susceptibility that may affect the survival of the species.

Because of the importance of this species as a commercial resource in the Adriatic Sea, those variations in macroscale parameters found in *C. gallina* shells could have economic implications for its fishery. More porous and less resistant to load fracture shells, found in warmer and more irradiated populations, are less resistant to breakage and could be more damaged during fishing with hydraulic dredges, with a larger amount of clams discarded from the trade. This could mean a higher catch effort for fishermen with a loss in economic yield.

In addition, the lower clam mass due to thinner and more porous shells in the southern populations, requires a major number of clams to obtain the same quantitative in kilograms compared to northern populations. Moreover, the northern populations could allocate a higher energy fraction to reinforce their shells at the expense of a lower somatic growth^29^. Contrarily, the warmer and more irradiated populations could allocate most of their assimilated energy towards somatic growth, compensating the negative aspect of shell variations with an increase in edible mass per catch and per kilogram, with a potential positive economical yield both for consumers and fishermen. Further analysis on edible animals may be necessary to understand if environmental parameters can affect the growth of soft tissue.

Despite the reported differences at the macroscale level, at the microscale and nanoscale levels, all populations of *C. gallina* showed the same skeletal features along the latitudinal gradient of SR and SST. The shells were always made of aragonite, as seen in other mollusks^51^,^52^,^53^. That aragonite always showed the same extent of atomic order^37^, evaluated by the grinding curve methodology. Indeed, grinding curves from *C. gallina* samples revealed that the aragonitic atomic order does not vary among populations along the SR an SST gradient. Those curves of *C. gallina* samples were close to that from the mollusk *Vermetus triqueter* shell and other aragonitic marine organisms (Cnidaria, *Balanophyllia europaea*, Anellida *Protula tubularia*). All of them were located between the synthetic, high order, and geogenic, low order, aragonìte curves^37^ (Supplementary Fig. S4). The invariance in nanoscale skeletal features is also confirmed by the constancy of shell chemical composition in the content Sr (FTIR data) and OM (TGA data). This is in agreement with what expected for *C. gallina*, a calcifying organism having a high degree of biological control over biomineralization, as other mollusks^7^,^38^.

Electron microscopy observations, at the micro(nano)scale level, showed that valves of *C. gallina* were characterized by two different aragonite structures: an external prismatic layer and an internal granular one, separated by a transition zone. The basic particles composing both layers were similar in shape, spheroidal, and size, below 1 μm. This was observed throughout the whole valve section, independently from the shell collection site. This constancy among all populations, suggests no difference among the aragonite “building blocks” at the micro and nanoscale level, a data that fits well with the results from spectroscopic (atomic order) and diffractometric (texture) measurements at nanoscale level.

Shell micro-density (density of the CaCO_3_ biomineral that composes the shell) did not change along the gradient. This information, together with the homogeneous amount of OM and Sr among populations, indicates that also other micro-skeletal parameters affecting micro-density, as occluded porosity and content of non crystalline CaCO_3_, did not change. The latter being linked to shell elasticity, a parameter at the macroscale level, constant along all populations of samples.

The constancy of OM content in *C. gallina* under different environmental conditions represents an important result, which adds relevance to the long recognized key role of OM in biomineral skeleton formation^7^,^8^,^54^,^55^,^56^. This despite the fact that still little information is known on varying OM content and composition in relation to environmental parameters.

As indicated by all micro and nanoscale analyses, the biomineral remained the same in all analyzed samples, indicating that the “building blocks” produced by the biomineralization process are substantially unaffected by the SR and SST variations among the different populations along the latitudinal gradient. Also the organization, morphology and packing, of the constituent mineral crystals were the same along the gradient. This constancy, as already discussed, has to be related to the high degree of biological control exerted by *C. gallina* over the calcification process, able to overcome environmental stresses^7^,^8^.

## Conclusions

Differences found in shell parameters of the clam *C. gallina* along the latitudinal gradient could be the outcome of phenotypic plasticity or a genetic adaptation of the populations subjected to different environmental parameters. Environmental parameters could directly affect shell morphology, such as temperature, or indirectly, influencing nutrient concentration and/or predator density. Shell morphology of the most irradiated and warmest populations was characterized by lighter, thinner, more porous and fragile shells, likely affecting the economic aspects of fisheries and the survival of the species. At the same time, populations of *C. gallina* did not show significant variations of structural parameters at the microscale and nanoscale level. The type of CaCO_3_ polymorph, the atomic order of the mineral skeletal phase and the percentage of organic matrix content were unaltered along the latitudinal gradient, indicating no effects of SR and SST on the building blocks produced by the biomineralization process of the clam shells.

## Materials and Methods

### Ethics Statement

This study was carried out following the fundamental ethical principles. According to the European normative that regulates minimum fishing size of *Chamelea gallina* (25 mm, Council Regulation (EC) No. 1967/2006), only clams of commercial size with minimum length of 25 mm were collected for this study.

### Collection and processing of specimens

Between August 2013 and April 2015, specimens of *C. gallina* of commercial size (25–30 mm), were collected from six sites along a latitudinal gradient in the Adriatic Sea from 45°42′N to 41°55′N (Supplementary Fig. S8).

Clams were sampled for each site using hydraulic dredges on soft bottoms in the subtidal zone at 3–7 m depth.

For each collected clam, the bivalve flesh was removed with a scalpel and the shell was cleaned with a toothbrush and washed with distilled water. The right and left valves were then separated, labeled and dried at 37 °C for one night to remove any moisture that may affect measurements.

### Shell biometric parameters

**Figure 4:**
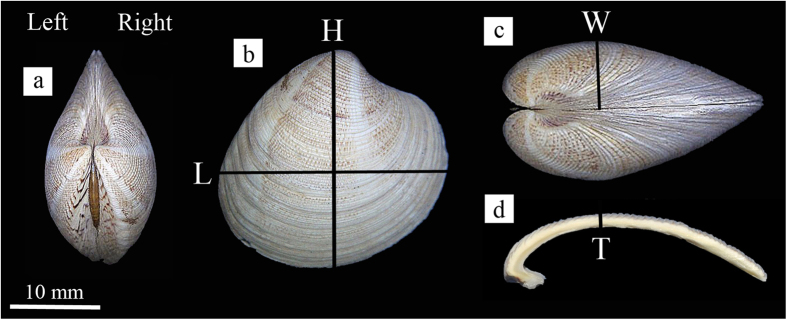
Shell parameters. (**a**) Frontal orientation; by placing the umbo upwards can be distinguished the valve left from the right one; (**b**) Lateral orientation, L = length, H = height; (**c**) cross-sectional orientation, W = width; (**d**) cross-section, T = thickness.

Clam shell length (maximum distance on the anterior-posterior axis), height (maximum distance on the dorsal-ventral axis; Fig. 4) and bi-dimensional shell shape parameters, were obtained with ImageJ software^57^ after data capture of each shell shape with a scanner (Acer Acerscan Prisa 620 ST 600 dpi, 0.04 mm/px). Bi-dimensional shell shape parameters, obtained from shell outline, were perimeter (the length of the outer contour of the shell), area (the space enclosed by the outer contour), aspect ratio (defined as length/height), solidity (area/convex area enclosed by the convex hull), circularity (with a value of 1 indicating a perfect circle and the value approaches 0 indicating an increasingly elongated shape) and roundness (the inverse of aspect ratio).Width (maximum distance on the lateral axis) and thickness (Fig. 4) of the valve were measured with a pair of calipers (±0.05 mm) and dry shell weight was measured using an analytical balance (±0.1 mg). Thickness was measured in the middle of each valve. Volume and shell density parameters were measured by means of the buoyant weight techniques, using a density determination kit Ohaus Explorer Pro balance (±0.1 mg; Ohaus Corp., Pine Brook, NJ, USA).

Measurements required for calculating shell parameters were:


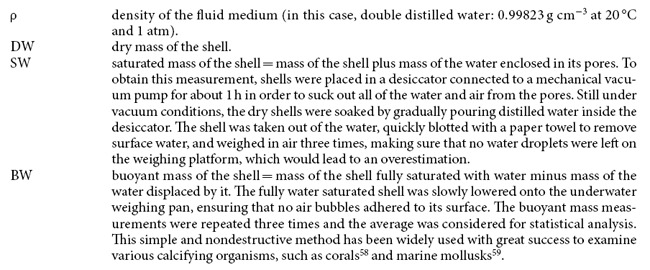



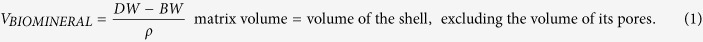



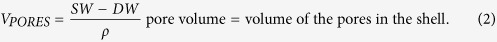



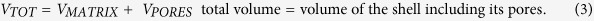


Additionally, the following skeletal parameters were calculated:


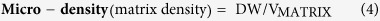



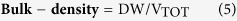



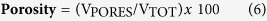


### Shell mechanical properties

To test for shell mechanical properties, compression tests were conducted using a universal testing machine equipped with a force transducer (Instron) of 1 kN maximum capacity. Thirty shell samples were randomly selected from each population and were brought to fracture load using a 3 cm diameter compression platen at a downward speed of 0.5 mm min^−1^. The Young’s modulus (kN mm^−1^) and the required force to fracture (Maximum load, kN) were recorded using the software Instron (Series IX).

Ten samples from each population were randomly selected and used for the following analyses. The valves were treated with a 5% sodium hypochlorite solution for three days to completely remove any trace of external skeletal organic tissue, and with a 1 M sodium hydroxide solution for one day to hydrolyze residual proteic materials from the shell surface. Samples were then rinsed with distilled water and dried at room temperature for one day. Subsequently, the left valve was sectioned with a dremel (300 series, Dremel System) from the umbo to the ventral margin. One half of each shell was finely ground in a mortar to obtain a homogenous powder to be used for diffractometric and spectroscopic analyses. A transversal section of about 3 mm in width was cut from the mid remaining shell for scanning electron microscope (SEM) observations (Supplementary Fig. S5a).

### Diffractometric measurements

XRD analyses were performed on five randomly selected specimens for each populations, preparing a thin, compact layer of powdered sample in a silica background signal free holder. Diffraction patterns for each sample were collected using an X’Celerator detector fitted on a PANalytical X’Pert Pro diffractometer, using Cu-Kα radiation generated at 40 kV and 40 mA. Data were collected within the 2θ range from 15° to 60° with a step size (Δ2θ) of 0.02° and a counting time of 1200 s. Fixed anti-scatter and divergence slits of 1/16° were used with 10 mm beam mask and all scans were carried out in ‘continuous’ mode. The XRD patterns were analyzed using the X’Pert HighScore Plus software (PANalytical) and the FWHM was measured for the peak (111) of each diffraction pattern.

### Spectroscopic measurements

FTIR analyses were performed on the same powder samples used for XRD. FTIR analyses were carried out using a FTIR Nicolet 380 Spectrometer (Thermo Electron Corporation) working in the range of wave-numbers 4000–400 cm^−1^ at a resolution of 2 cm^−1^. Sample disks were obtained mixing a small amount (1 mg) of finely ground sample with 100 mg of KBr and applying a pressure of 670.2 MPa to the mixture using a hydraulic press. For each spectrum, characteristic CaCO_3_ active vibrational modes *ν*_*2*_, *ν*_*3*_ and *ν*_*4*_ bands were identified and their intensities were measured. To compare atomic order among the six populations, for a sample of each population the intensity of the *ν*_*2*_ and *ν*_*4*_ bands were normalized to the *ν*_*3*_ band and then graphed after successive grinding processes^37^. To understand if there were differences in the atomic order within crystals, the plotted curves of *C. gallina* were compared with grinding curves of other calcifying marine organisms (*Balanophyllia europaea, Protula tubularia, Vermetus triqueter*) and geogenic and synthetic aragonite^37^.

### Evaluation of OM content

TGA was performed to estimate the OM content of each shell, using an SDT Q600 instrument (TA Instruments). Powdered samples (5–10 mg) from 5 to 10 valves for each site were placed in a ceramic crucible. The analyses were performed under a nitrogen flow of 100 mL/min with a first heating ramp from 30 to 120 °C at 10 °C min^−1^ heating rate, an isothermal at 120° for 5 min, and a second heating ramp from 120 to 600 °C, at 10 °C min^−1^ heating rate.

### Skeletal micro-textural analyses

SEM observations were carried out on a subset of individuals from Chioggia and Capoiale (characterized respectively by lowest and highest SST values), to obtain representative information on the textural characteristics of *C. gallina* shell. Skeletal features were investigated on the transversal valve sections. Each section was etched with an acetic acid solution (1% *v*/*v*) for 1 minute to remove debris and artifacts from cutting. Samples were coated with a gold layer (5 nm) and analyzed with a SEM Hitachi S4000.

### Environmental parameters

SR (W m^−2^) and SST (°C) data were obtained for each site from the Euro-Mediterranean Center on Climate Change (CMCC http://oceanlab.cmcc.it/afs/) data banks. Mean annual SR and SST were calculated from daily values measured from July 2011 to June 2015 (number of daily values = 1447 for each site), to enclose the almost full lifespan of two-three years for each sample.

### Statistical analyses

Levene’s test was used for testing homogeneity of variance and Kolmogorov-Smirnov’s test was used for testing normality of variance for both environmental and shell parameters. One-way analysis of variance (ANOVA) was used to test the significance of the differences among sites for environmental variables and shell parameters. When assumptions for parametric statistics were not fulfilled, the non-parametric Kruskal-Wallis equality-of-populations rank test was used instead. Student’s t test was used to compare the mean right and left valve shell parameters (length, width, height, thickness, mass, volume, micro-density, bulk density and porosity) in each site. Spearman’s rank correlation coefficient was used to calculate the significance of the correlations between shell parameters and environmental parameters. Spearman’s rank correlation coefficient is an alternative to Pearson’s correlation coefficient; it is useful for data that are non-normally distributed and do not meet the assumptions of Pearson’s correlation coefficient^60^. All analyses were computed using PASW Statistics 22.0 (Apache Computer Software Foundation, Forest Hill, USA). Principal component analysis (PCA) was used to test the bi-dimensional shell shape among populations using R Studio software (see Supplementary Information).

## Additional Information

**How to cite this article:** Gizzi, F. *et al.* Shell properties of commercial clam *Chamelea gallina* are influenced by temperature and solar radiation along a wide latitudinal gradient. *Sci. Rep.* **6**, 36420; doi: 10.1038/srep36420 (2016).

**Publisher’s note**: Springer Nature remains neutral with regard to jurisdictional claims in published maps and institutional affiliations.

## Supplementary Material

Supplementary Information

## Figures and Tables

**Table 1:** Environmental parameters.

Code	Latitude (°)	n	SR (W m^−2^)		SST (°C)	
			mean (SE)	Range	mean (SE)	Range
MO	45.7	1447	159.4 (2.5)	154.4–164.4	16.96 (0.19)	16.58–17.35
CH	45.2	1447	160.8 (2.5)	155.8–165.7	16.47 (0.19)	16.09–16.84
GO	44.8	1447	163.8 (2.6)	158.7–168.8	16.54 (0.19)	16.17–16.92
CE	44.2	1447	165.2 (2.5)	160.2–170.2	17.05 (0.20)	16.65–17.45
SB	43.1	1447	172.4 (2.5)	167.4–177.4	17.90 (0.19)	17.52–18.28
CA	41.9	1447	180.4 (2.6)	175.4–185.5	18.60 (0.17)	18.27–18.93

Mean annual values for solar radiation (SR) and sea surface temperature (SST) from 2011 to 2014, of the sites. n = number of collected data; SE = standard error.

Values for each population, in decreasing order of latitude: MO (Monfalcone), CH (Chioggia), GO (Goro), CE (Cesenatico), SB (San Benedetto), CA (Capoiale).

**Table 2:** Shell biometric parameters.

Code	n	Length (mm) mean (CI)	Height (mm) mean (CI)	Width (mm) mean (CI)	Thickness (mm) mean (CI)	Mass (g) mean (CI)	Volume (cm3) mean (CI)
MO	40	26.85 (0.40)	22.28 (0.27)	7.41 (0.13)	1.60 (0.05)	2.15 (0.07)	0.77 (0.03)
CH	40	26.14 (0.41)	21.45 (0.32)	6.39 (0.09)	1.33 (0.06)	1.66 (0.07)	0.59 (0.02)
GO	40	26.06 (0.47)	21.29 (0.39)	6.69 (0.13)	1.26 (0.05)	1.59 (0.08)	0.57 (0.03)
CE	40	26.52 (0.49)	21.49 (0.39)	6.47 (0.15)	1.17 (0.04)	1.57 (0.09)	0.56 (0.03)
SB	40	26.39 (0.45)	21.71 (0.32)	6.44 (0.11)	1.14 (0.04)	1.52 (0.07)	0.54 (0.02)
CA	40	26.40 (0.51)	21.42 (0.42)	6.13 (0.12)	1.06 (0.03)	1.41 (0.08)	0.51 (0.03)
K-W		NS	***	***	***	***	***

Macroscale level. Average of biometric parameters at each site. n = number of samples; CI = 95% confidence interval.

Populations are arranged in order of decreasing latitude: MO (Monfalcone), CH (Chioggia), GO (Goro), CE (Cesenatico), SB (San Benedetto), CA (Capoiale). K-W = Kruskal-Wallis equality-of-populations rank test, NS = not significant, ***p < 0.001.

**Table 3:** Shell biometric parameters.

Code	n	Micro-density (g cm^−3^) mean (CI)	Bulk density (g cm^−3^) mean (CI)	Apparent porosity (%) mean (CI)
MO	40	2.80 (0.010)	2.72 (0.010)	2.86 (0.149)
CH	40	2.82 (0.010)	2.72 (0.012)	3.30 (0.317)
GO	40	2.81 (0.012)	2.70 (0.013)	3.87 (0.261)
CE	40	2.81 (0.003)	2.70 (0.011)	4.04 (0.342)
SB	40	2.82 (0.006)	2.70 (0.008)	3.98 (0.343)
CA	40	2.80 (0.004)	2.68 (0.008)	4.20 (0.312)
K-W		NS	***	***

Macro- and microscale levels. Micro-density, bulk density and apparent porosity of the sites in decreasing order of latitude. n = number of samples; CI = 95% confidence interval.

Populations are arranged in order of decreasing latitude: MO (Monfalcone), CH (Chioggia), GO (Goro), CE (Cesenatico), SB (San Benedetto), CA (Capoiale). K-W = Kruskal-Wallis equality-of-populations rank test, ***p < 0.001.

**Table 4:** Shell mechanical properties.

Code	n	Young’s modulus (kN mm^−1^) mean (CI)	Maximum Load (kN), mean (CI)
MO	14	2.50 (0.54)	0.13 (0.02)
CH	30	2.80 (0.16)	0.15 (0.01)
GO	28	2.35 (0.18)	0.11 (0.01)
CE	21	2.61 (0.13)	0.13 (0.02)
SB	28	2.30 (0.19)	0.11 (0.02)
CA	20	2.18 (0.29)	0.09 (0.01)
K-W		***	***

Young’s modulus (kN mm^−1^) and Maximum load (kN) mean value for each population. n = number of samples; CI = 95% confidence interval.

Values for each population, in decreasing order of latitude: MO (Monfalcone), CH (Chioggia), GO (Goro), CE (Cesenatico), SB (San Benedetto), CA (Capoiale). K-W = Kruskal-Wallis equality-of-populations rank test, ***p < 0.001.
