# Supplementary material for: Shell properties of commercial clam Chamelea gallina are influenced by temperature and solar radiation along a wide latitudinal gradient
Source: Sci Rep. 2016 Nov 2;6:36420. doi: 10.1038/srep36420 (PMC5090357; doi:10.1038/srep36420)
Supplement: Supplementary Information [file srep36420-s1.pdf]

**Supplementary information**

# Shell properties of commercial clam *Chamelea gallina* are influenced by temperature and solar radiation along a wide latitudinal gradient

Francesca Gizzi[1], Maria Giulia Caccia[1], Ginevra Allegra Simoncini[1], Arianna Mancuso[1,2], Michela Reggi[3], Simona Fermani[3], Leonardo Brizi[4,5], Paola Fantazzini[4,5], Marco Stagioni[2], Giuseppe Falini[3*], Corrado Piccinetti[2*], Stefano Goffredo[1*]

## Tables

**Table S1. Textural data of biogenic aragonite.** Full width at half maximum (FWHM) of the X-ray powder diffraction peak (111) and normalized (over $\nu_3$) intensities of the aragonite Fourier transform infrared (FTIR) bands $\nu_2$ and $\nu_4$. The angle (2θ) of the maximum of the diffraction peaks and wavenumbers of the maximum of the FTIR bands do not change among samples. n = number of samples; CI = 95% confidence interval. $\nu_2$, $\nu_3$ and $\nu_4$ = characteristic calcium carbonate active vibrational modes.

| Code | n | FWHM mean (CI) | $\nu_2/\nu_3$ mean (CI) | $\nu_4/\nu_3$ mean (CI) |
|---|---|---|---|---|
| MO | 5 | 0.170 (0) | 0.386 (0.014) | 0.095 (0.005) |
| CH | 5 | 0.180 (0) | 0.345 (0.009) | 0.058 (0.012) |
| GO | 5 | 0.180 (0.006) | 0.401 (0.014) | 0.102 (0.015) |
| CE | 5 | 0.172 (0.004) | 0.388 (0.009) | 0.100 (0.008) |
| SB | 5 | 0.174 (0.005) | 0.352 (0.014) | 0.090 (0.007) |
| CA | 5 | 0.188 (0.004) | 0.369 (0.011) | 0.075 (0.005) |
| K-W | | *** | | |

Values for each population, in decreasing order of latitude: MO (Monfalcone), CH (Chioggia), GO (Goro), CE (Cesenatico), SB (San Benedetto) and CA (Capoiale). K-W = Kruskal-Wallis equality-of-populations rank test, *** = $p < 0.001$.

**Table S2. Intra-skeletal organic matrix content.** Mean organic matrix (OM) percentage weight loss for each population of *C. gallina.* n = number of samples; CI = 95% confidence interval.

| Code | n | OM (%) mean (CI) |
|---|---|---|
| MO | 10 | 1.95 (0.09) |
| CH | 10 | 1.86 (0.11) |
| GO | 5 | 1.83 (0.16) |
| CE | 5 | 1.93 (0.16) |
| SB | 5 | 1.72 (0.13) |
| CA | 10 | 1.97 (0.08) |
| K-W | | NS |

Values for each population, in decreasing order of latitude: MO (Monfalcone), CH (Chioggia), GO (Goro), CE (Cesenatico), SB (San Benedetto) and CA (Capoiale). K-W = Kruskal-Wallis equality-of-populations rank test, NS = not significant

**PCA analysis**

PCA analysis was obtained including bi-dimensional shell shape parameters, (perimeter, area, aspect ratio, solidity, circularity and roundness), and length and height, to observe the possible differences in bi-dimensional shell shape among populations, using R studio software[1]. Length, height, perimeter and area were related to the PC1, aspect ratio and roundness were related to the PC2, and together explained the 72.9% of the variance (Supplementary Fig. S2a). Circularity and solidity, closer to the centre of the plot, were related to the PC3, which was not considered in the analysis because of the low variance explained (18.7%; Supplementary Fig. S2a). PCA analysis resulted in a similar bi-dimensional outline shape in shells of *C. gallina* from six populations along the Italian Adriatic coast, due to the overlapping position of dots in the space (Supplementary Fig. S2b).

The result found using traditional morphometric analysis (PCA), was compared with the result of Palmer et al.[2], that studied the bi-dimensional shape of Mediterranean specimens of *C. gallina* by combining elliptic Fourier decomposition of the shell perimeter and canonical variate analysis. They find a geographical variability in bi-dimensional shell shape of *C. gallina*, by comparing samples from seven geographical groups with highly various environments, from Màlaga, Spain, to Venice, Italy[2]. The differences in bi-dimensional shell shape of *C. gallina* found by Palmer et al.[2] is not in contrast with our result, because we considered six population located along ~400 km in the Adriatic Sea, from Monfalcone (MO) to Capoiale (CA) and characterized by more similar environmental conditions. Nevertheless, differences in *C. gallina* shells among populations were obtained analysing the tri-dimensional shell biometric parameters (length, height, width, mass, volume, bulk-density and apparent porosity) not considered in Palmer et al.[2], but considered in this study (see main text).

Analysing both, bi-dimensional shape shell parameters and tri-dimensional biometric shell parameters, it was possible to discriminate groups rather geographically close and revealed which shell parameters differed among populations.

**Figures**

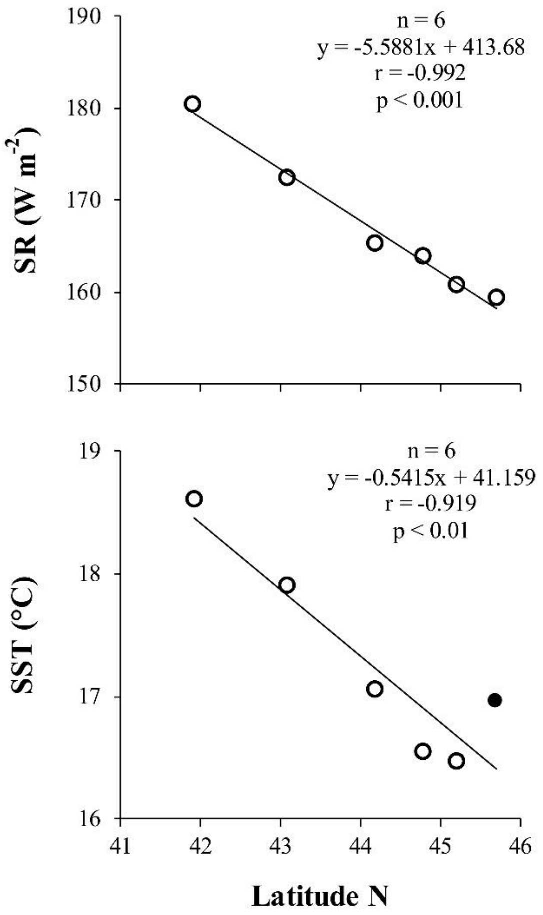


**Figure S1**. **Environmental parameters.** Relationship between environmental parameters (mean annual SR and SST) and the latitude of study sites along the coast of Italy. The black dot indicates the site of Monfalcone, which was characterized by higher temperature than expected at its latitude. n = number of stations; r = Pearson's correlation coefficient.

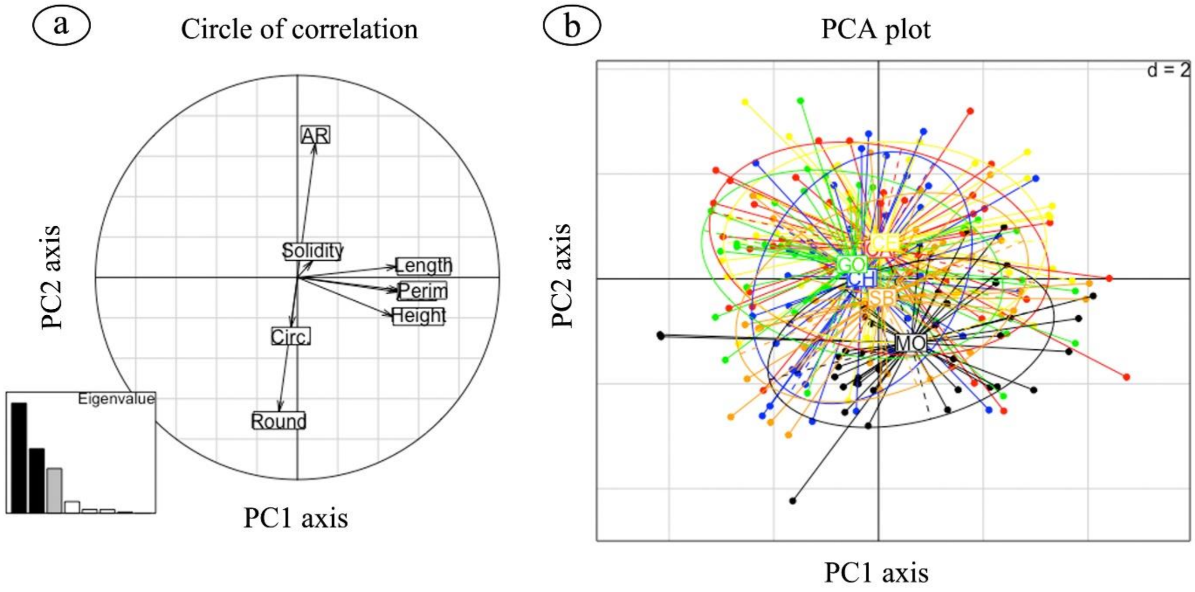


**Figure S2. PCA analysis.** (a) Circle of correlations between the bi-dimensional shell shape parameters and length and height and the first two principal components (PC1, PC2). Eigenvalues plot shows the percentage of explained variance for each component (PC1=46 %, PC2=26.9%, PC3=18.7%). Length, perimeter (Perim), height and area were related to PC1; the area variable is not visible, because covered by perimeter. Roundness (Round) and aspect ratio (AR) were related to PC2; solidity and circularity (Circ) were related to PC3. (b) PCA plot of the distribution of the dots in the space related to the bi-dimensional shell shape parameters and length and height, interpreted by PC1 and PC2. Each dot represents one *C. gallina* shell. MO (Monfalcone) in black; CH (Chioggia) in blue; GO (Goro) in green; CE (Cesenatico) in yellow; SB (San Benedetto) in orange; CA (Capoiale) in red.

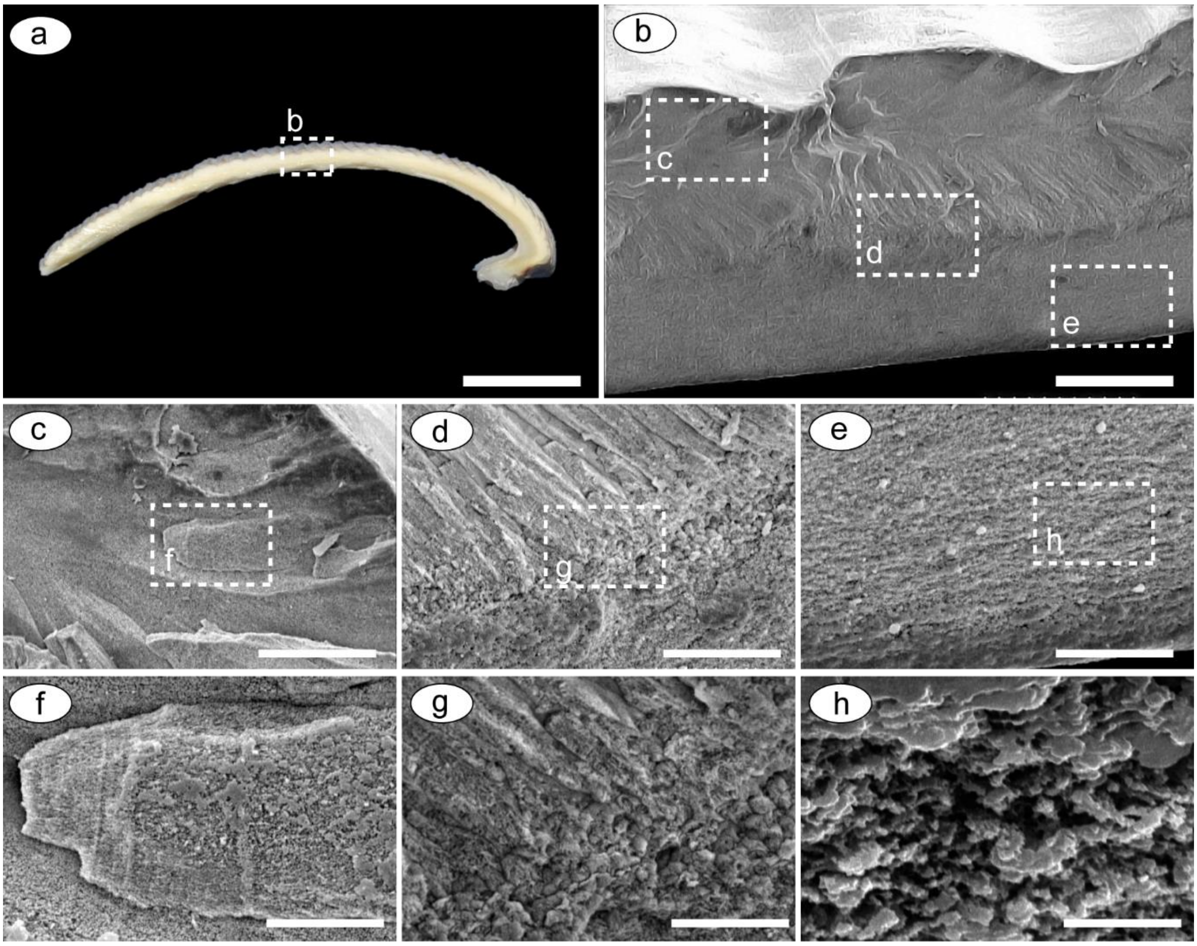


**Figure S3. Scanning electron microscopy images.** Skeletal morphology of *C. gallina* microstructure. The reported images are representative of all observed valves. (a, b) Images of the entire valve section. (a) Scale bar 5 mm. (b) Scale bar 0.3 mm. (c, d, e) Outer layer, transition layer and inner layer, respectively. Scale bars: 100, 50 and 50 μm, respectively. (f, g, h) Details of the outer layer, transition layer and inner layer, respectively. Scale bar 30, 20 and 5 μm, respectively. Outlined rectangles indicate areas of interest subject to higher magnifications in subsequent images.

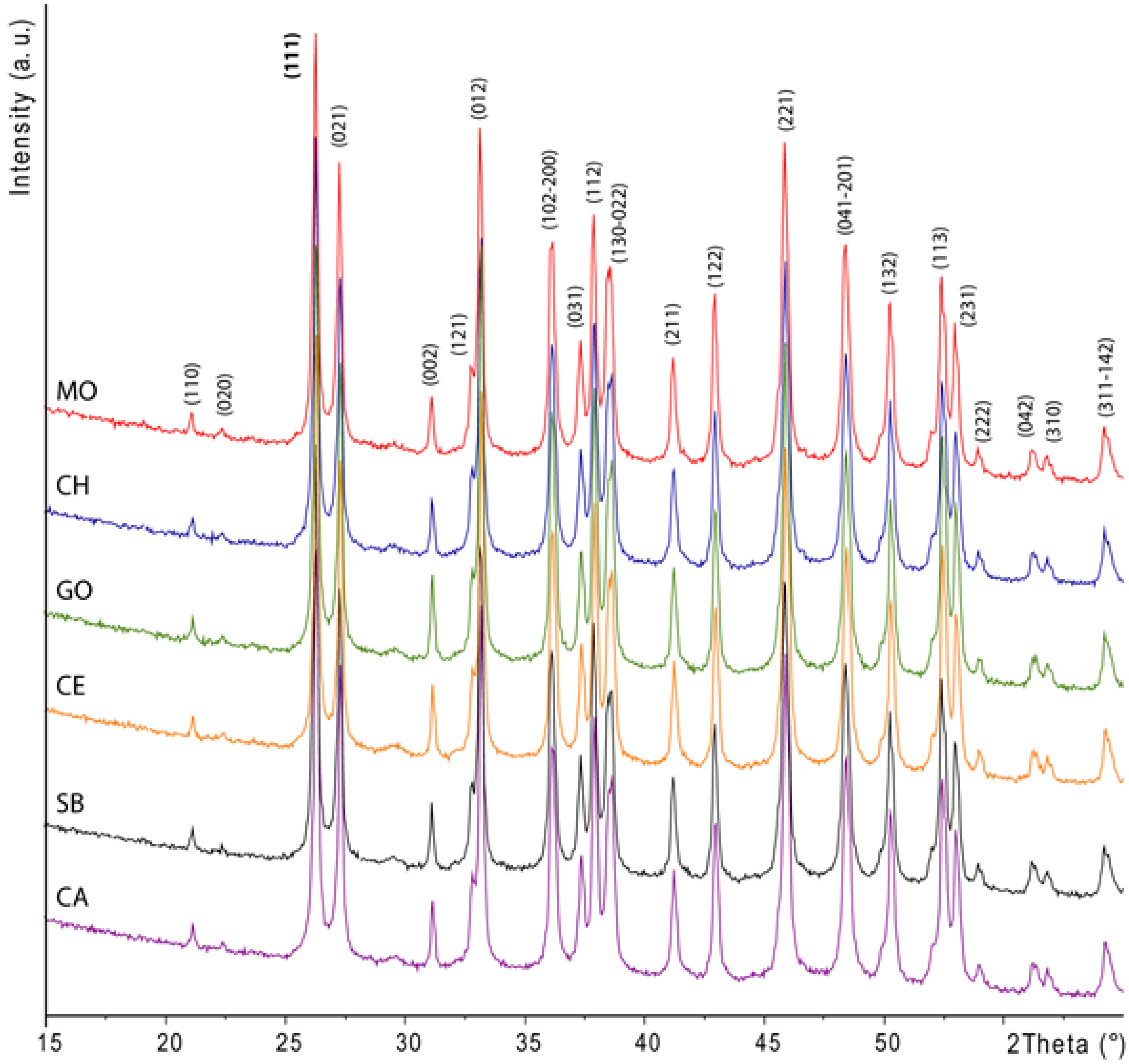


**Figure S4. X-ray powder diffraction (XRD) patterns from ground shells of *C. gallina*.** 5 diffractograms for each population were obtained. A representative diffraction pattern is shown for each population, in decreasing order of latitude: MO (Monfalcone), CH (Chioggia), GO (Goro), CE (Cesenatico), SB (San Benedetto) and CA (Capoiale). The diffraction peaks were labeled with the Miller index according to the PDF 01-075-2230[3]. All the peaks were assigned to aragonite. Diffraction patterns are offset to increase readability.

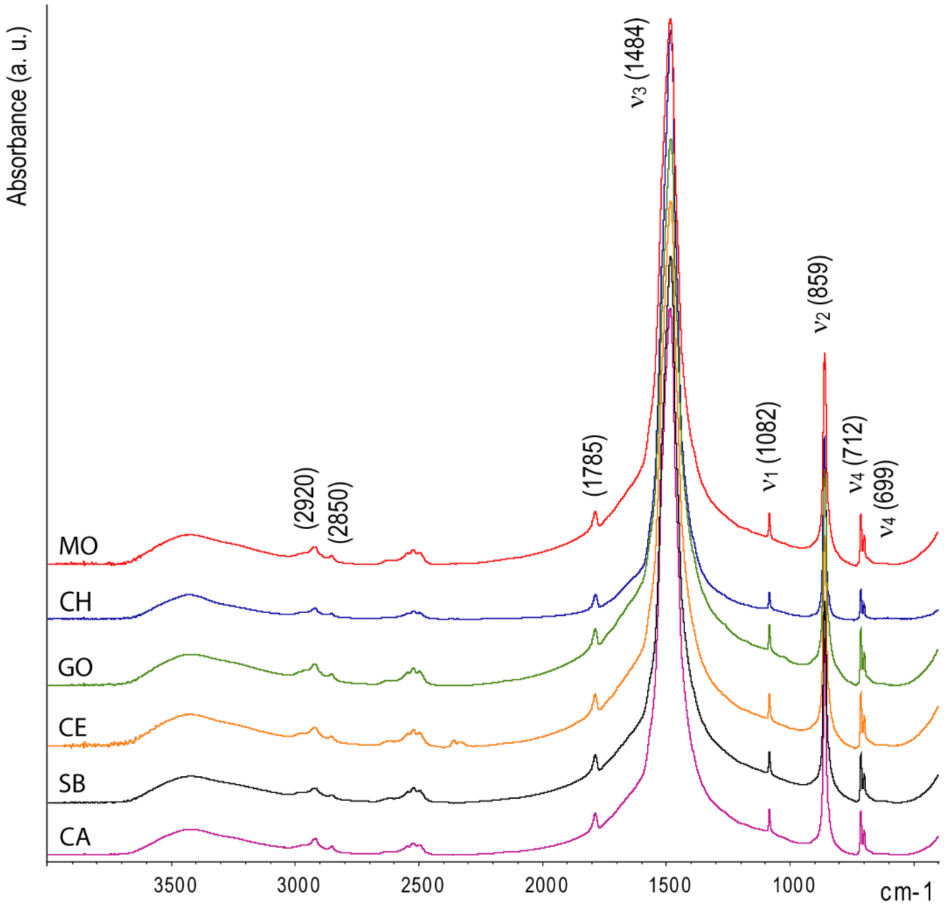


**Figure S5. Fourier transform infrared (FTIR) spectra from ground shells of *C. gallina*.** 5 spectra for each population were obtained. A representative FTIR spectrum is shown for each population, in decreasing order of latitude: MO (Monfalcone), CH (Chioggia), GO (Goro), CE (Cesenatico), SB (San Benedetto) and CA (Capoiale). Wavenumbers of the main absorption bands are indicated and those due to aragonite are assigned. Spectra are offset to increase readability.

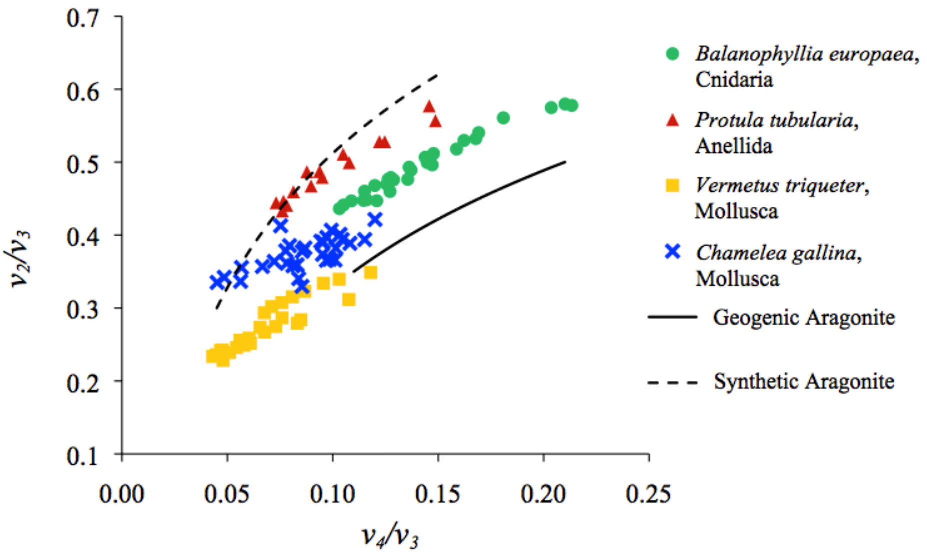


**Figure S6. Evaluation of atomic order by FTIR grinding curves.** Combined plots of the $\nu_4/\nu_3$ vs. $\nu_2/\nu_3$ band heights of *C. gallina* from this study, *B. europaea*, *P. tubularia* and *V. triqueter*[4] and fitted curves of the $\nu_4/\nu_3$ vs. $\nu_2/\nu_3$ peak heights of geogenic and synthetic aragonite[5].

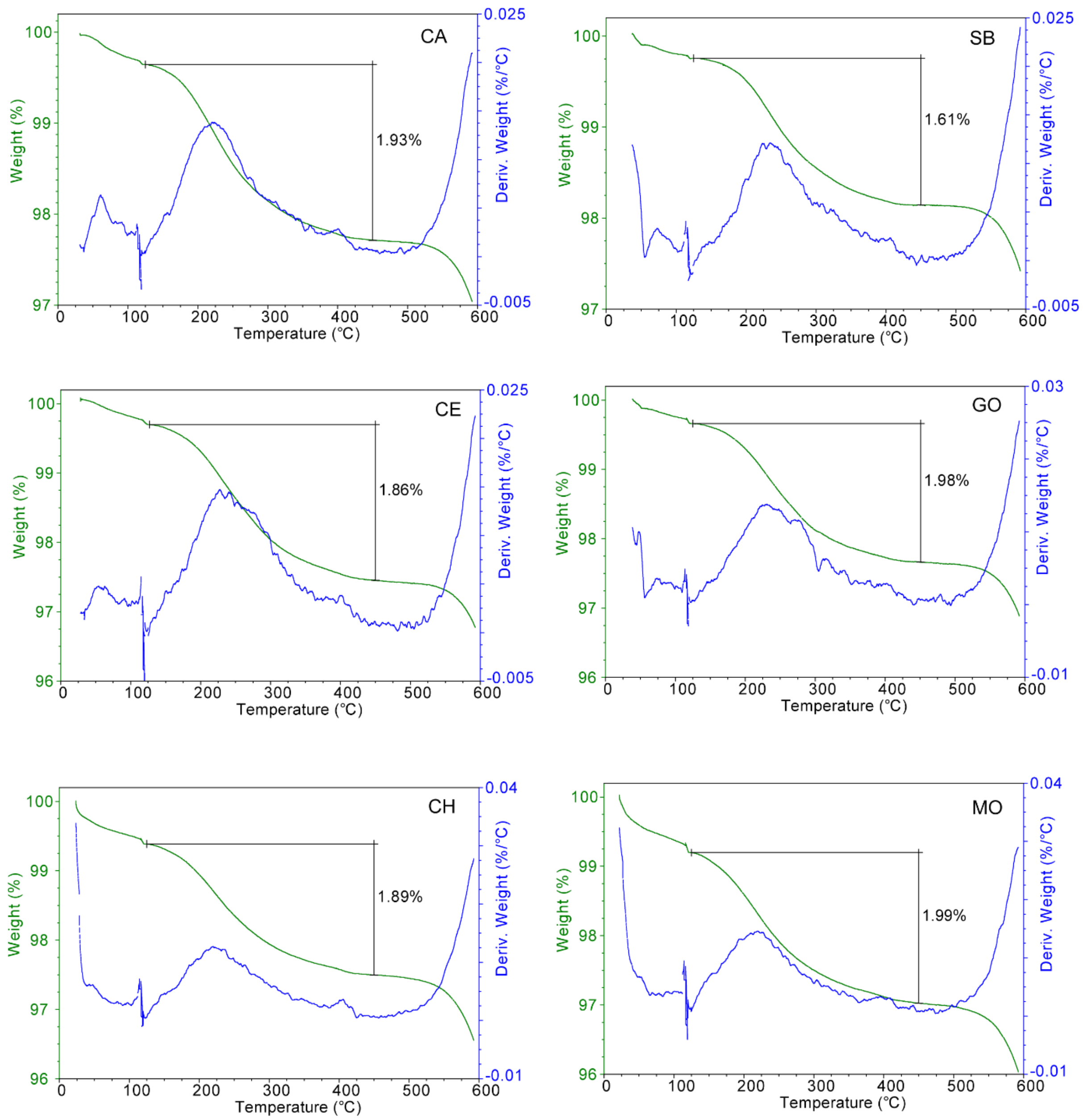


**Figure S7. Intra-skeletal organic matrix content.** A representative thermo gravimetrical profile is shown for each population, in increasing order of latitude: CA (Capoiale), SB (San Benedetto), CE (Cesenatico), GO (Goro), CH (Chioggia) and MO (Monfalcone). Weight lost (%) and derivate weight lost (%/°C) profiles are drown with black and blue lines, respectively.

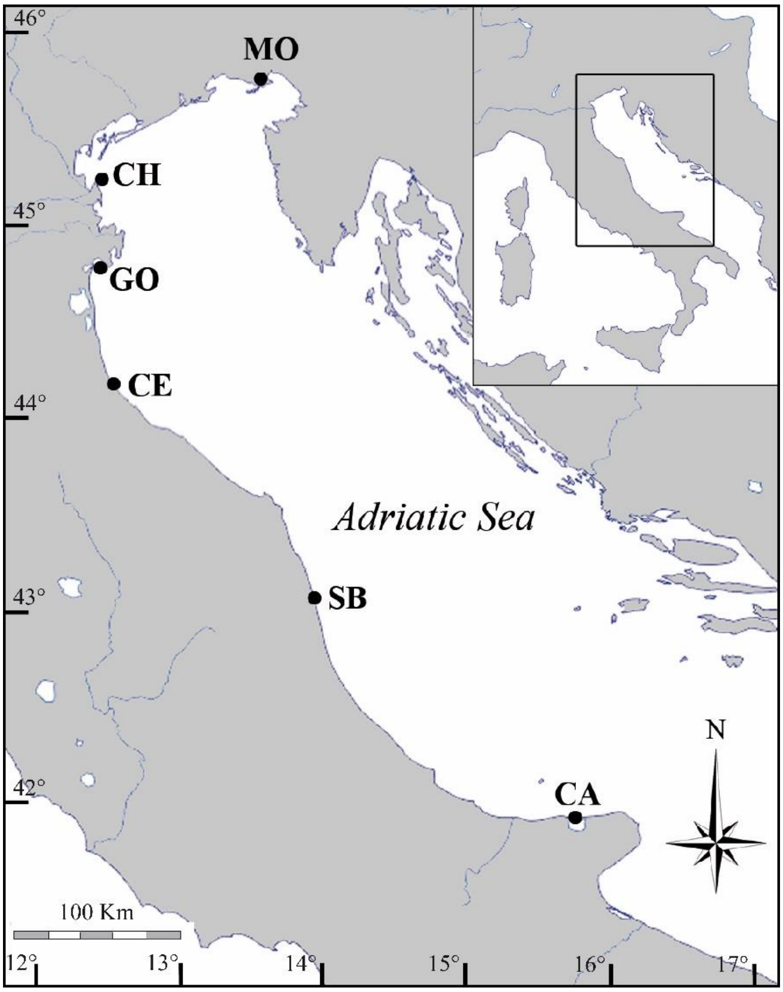


**Figure S8. Map of the Adriatic coastline indicating the sites where the *C. gallina* clams were collected.** Abbreviations and coordinates of the sites in decreasing order of latitude: MO, Monfalcone 45°42′N, 13°14′E; CH, Chioggia 45°12′N, 12°19′E; GO, Goro 44°47′N, 12°25′E; CE, Cesenatico 44°11′N, 12°26′E; SB, San Benedetto 43°5′N, 13°51′E; CA, Capoiale 41°55′N, 15°39′E. The map was downloaded from d-maps.com site (http://www.d-maps.com/carte.php?num_car=5993&lang=it) and modified with Adobe Photoshop CS4.

**Supplementary references**


1. Dray, S. & Dufour, A. B. The ade4 package: implementing the duality diagram for ecologists. *J. Stat. Softw.* **22,** 1-20 (2007).

2. Palmer, M., Pons, G. X. & Linde, M. Discriminating between geographical groups of a Mediterranean commercial clam (*Chamelea gallina* (L.): Veneridae) by shape analysis. *Fish. Res.* **67,** 93-98 (2004).

3. Jarosch, D. & Heger, G. Neutron diffraction refinement of the crystal structure of aragonite. *Tschermaks Mineral. Petrogr. Mitt*. **35,** 127-131 (1986).

4. Sabbioni, L. *Influence of Global Warming on Marine Calcifying Organisms.* MS Thesis, Department of Chemistry "Giacomo Ciamician" (University of Bologna, Italy, 2012).

5. Suzuki, M., Dauphin, Y., Addadi, L. & Weiner, S. Atomic order of aragonite crystals formed by mollusks. *Cryst. Eng. Comm.* **13,** 6780-6786 (2011).
